# Inhaler identification: evaluating a potential screening method for adherence in chronic respiratory disease management

**DOI:** 10.36416/1806-3756/e20250393

**Published:** 2025-07-22

**Authors:** David Silva Gomes, Carlos Seixas, João Cravo

**Affiliations:** 1. Departamento de Pneumologia do Hospital Infante D. Pedro, Unidade Local de Saúde da Região de Aveiro, Aveiro, Portugal.; 2. Portucalense University, Research on Economics, Management and Information Technologies, REMIT, Porto, Portugal.; 3. Centro de Economia e Finanças, Universidade do Porto, Porto, Portugal.

**Keywords:** COPD, asthma, inhalers, therapeutic adherence, visual perception

## Abstract

**Objectives:**

This study explores the relationship between inhaler visual identification, naming, and adherence outcomes, and evaluates the potential of combining these factors into a screening tool for identifying poor adherence.

**Methods:**

This observational, prospective study included adult patients with COPD, asthma, or asthma+COPD who had been on chronic inhalation therapy for at least the past year. Data were collected through patient interviews and medical records. Adherence was assessed using the Test of Adherence to Inhalers (TAI) questionnaire and prescription records, calculated as the Proportion of Days Covered (PDC). The patients completed a questionnaire to evaluate their ability to visually identify and name their inhalers.

**Results:**

Among the 196 participants, significant differences in adherence levels were observed across the COPD, asthma, and asthma+COPD groups, with COPD patients demonstrating higher adherence rates (p=0.001). Concordance between TAI and PDC was highest in the COPD group (75.0%), compared to the asthma (51.3%) and asthma+COPD (55.5%) groups. Correct naming of inhalers was not significantly correlated with adherence. However, correct inhaler visual identification was associated with better adherence. Incorrect visual identification showed low sensitivity (15.9%) but high specificity (92.6%) for detecting poorly adherent patients.

**Conclusions:**

The ability to visually identify inhalers was associated with better adherence, while the ability to name inhalers was not. Although incorrect visual identification has limited utility as a screening tool, it may still serve as a rapid and practical method for identifying poorly adherent patients in clinical practice.

## INTRODUCTION

Chronic obstructive pulmonary disease (COPD) and asthma are prevalent respiratory conditions that significantly impact patients’ quality of life and healthcare systems worldwide.^1^ ^,^ ^2^ Adherence to inhaler therapy is a critical component in effectively managing these disorders. Poor adherence can lead to suboptimal disease control, increased healthcare utilization, reduced quality of life, and higher mortality rates in these patients.^3^ ^-^ ^11^

Assessing adherence during medical consultations is crucial but often challenging and time-consuming. Indirect methods such as patient self-reporting, prescription records, and electronic monitoring are commonly used.^12^ Self-reporting is frequently employed in clinical practice but tends to overestimate adherence due to its subjective nature.^13^ Prescription records are generally less biased but may be more time-consuming for clinicians to analyze. Electronic monitoring, while promising, is not yet widely accessible. Consequently, there is a need for a quick and practical initial screening tool to identify patients who may benefit from more comprehensive adherence assessments.

Various determinants that influence adherence, either increasing or decreasing its likelihood, have already been identified in patients with COPD and/or asthma.^14^ ^-^ ^16^ Nevertheless, there is limited evidence regarding the impact of correctly identifying and naming inhalers on adherence to chronic inhalation therapy.

The aim of this study, which was conducted within the Pulmonology Department of our hospital, was to explore the relationship between inhaler visual identification and naming and adherence outcomes, as well as to evaluate the potential of combining these factors as a screening tool for detecting poor adherence.

This study investigated adherence to chronic inhalation therapy among patients with COPD, asthma, and asthma+COPD by combining self-reporting and objective adherence measures to provide a comprehensive analysis of adherence patterns and their predictors.

Through this approach, we aim to deepen the understanding of adherence behaviors in chronic respiratory conditions and propose a rapid, practical tool for assessing adherence in clinical settings. Ultimately, our goal is to contribute to improved disease management and better patient outcomes.

## METHODS

### Study Design and Participants

This analytical, observational, cross-sectional prospective study was conducted in the Pulmonology Department of our hospital. Adult patients who attended consultations or were hospitalized there between January 2022 and July 2023 were considered for inclusion. Eligible patients were required to have a diagnosis of chronic obstructive pulmonary disease (COPD), asthma, or asthma+COPD, as determined by the attending physician, and to have been undergoing chronic inhalation therapy for at least the past year. Individuals with visual or cognitive impairments that could interfere with their ability to adequately complete the questionnaires were excluded.

### Data Collection

Data were collected through patient interviews and medical record reviews. The diagnosis of COPD, asthma, or asthma+COPD was confirmed by the attending pulmonologist. Relevant clinical and demographic information, including details on chronic inhalation therapy and its duration, was recorded. Adherence to chronic inhalation therapy was assessed using two distinct methods: patient self-report via the Test of Adherence to Inhalers (TAI) questionnaire, and prescription records, by calculating the Proportion of Days Covered (PDC). Visual identification and naming of the patient’s inhaler(s) were evaluated using a questionnaire developed by the authors for this purpose (see Supplementary Material). Correct naming was defined as the patient’s ability to provide either the commercial name or the pharmaceutical components of their inhaler(s).

### Chronic Inhalation Therapy Adherence

As previously mentioned, adherence to chronic inhalation therapy was assessed using two methods, patient self-report and review of prescription records, as detailed below:

- Patient Self-Report: The TAI questionnaire was developed to assess adherence to inhaler use among patients with respiratory conditions such as asthma and COPD. It is designed to identify patterns of non-adherence and their underlying causes, thereby facilitating targeted interventions to improve medication use. The TAI consists of two parts: a 10-item patient self-report questionnaire (TAI-10) and a 12-item healthcare professional (HCP) assessment. Each item on the TAI-10 is scored from 1 to 5, with higher scores indicating better adherence. The total score ranges from 10 to 50 and categorizes adherence into three levels: good adherence (50), intermediate adherence (46-49), and poor adherence (≤ 45).^17^ In this study, we used the 10-item TAI questionnaire, which has been validated for the Portuguese population.

- Prescription Records Review: Adherence was also assessed using the PDC, calculated based on prescription refill data. The PDC estimates the proportion of time a patient has had access to their medication, providing an objective measure of medication availability. Unlike self-reported tools such as the TAI, the PDC is less susceptible to recall bias or social desirability. Although it assumes that dispensed medication is actually taken-an acknowledged limitation-the PDC is generally considered a more specific and objective measure of adherence and is often used as a reference standard in studies on chronic medication use. The PDC was calculated using the following formula:

$$\;PDC\;=\left(\frac{\;Number\;of\;days\;\mathrm{cov}ered\;by\;medication\;}{\;Number\;of\;days\;under\;observation\;}\right)\times 100$$

In this study, the observation period was one year (365 days), and medication coverage was determined using prescription refill data from the national prescription platform PEM (*Prescrição Eletrónica de Medicamentos*). A PDC of 80% or higher was considered indicative of good adherence.^18^ ^,^ ^19^

### Questionnaire

The patients were asked to complete a questionnaire to assess their ability to visually identify and name their inhaler(s) (see Supplementary Material). They were shown a set of images depicting all inhalation devices available on the Portuguese national market and asked to select the image(s) corresponding to their prescribed inhaler(s). The physician recorded the selected images. Next, the patients were asked to name either the commercial designation or the pharmaceutical components of their inhaler(s). Finally, the physician recorded whether the patient correctly or incorrectly identified and named their inhaler(s).

### Statistical Analysis

Data were analyzed using Stata Statistical Software (StataCorp. 2023. Stata Statistical Software: Release 17. College Station, TX, USA: StataCorp LLC). Categorical variables were presented as frequencies and percentages, while normally distributed continuous variables were expressed as mean ± standard deviation (SD). The chi-square test was used to compare categorical variables. For continuous variables, an independent-samples t-test was applied when the data followed normal distribution, and Mann-Whitney U tests were used for skewed distributions. Pearson’s correlation was used to assess the relationship between TAI scores and PDC. Concordance between the two adherence measures was also calculated. To align the three-level TAI classification with the binary PDC categorization, TAI scores indicating “intermediate adherence” were grouped with “good adherence”, forming a single “good adherence” category. This approach enabled direct comparison between the tools and is consistent with a previous study that considers intermediate TAI scores to reflect acceptable adherence levels.^17^

A fractional logit model analysis was conducted to identify predictors of adherence to chronic inhalation therapy. Although a fractional probit model yielded similar results, it demonstrated a poorer fit to the data. Fractional logit models are appropriate for dependent variables expressed as proportions or bounded between 0 and 1. Therefore, the TAI score was transformed to fit this model using the following formula:

$$\frac{TAI-10}{50-10}$$

No transformation was necessary for the PDC. A p-value of <0.05 was considered statistically significant.

### Ethical Considerations

This study was approved by our hospital’s Ethics Committee (Reference No. 215-CA-2-7). Informed consent was obtained from all participants prior to their inclusion.

## RESULTS

A total of 196 participants were included. The general characteristics of the study sample and their adherence to chronic inhalation therapy are presented in Table 1.

**Table 1:** General characteristics and characterization of adherence to chronic inhalation therapy in the study sample.

Characteristics	COPD (n = 104)	Asthma (n = 74)	Asthma+COPD (n = 18)	All (n = 196)	p-value
Age (years)	72.1 ± 9.3	58.5 ± 17.7	63.4 ± 4.6	66.2 ± 14.6	0.000
Sex, n (%)					0.000
> Female	26 (25.0%)	62 (83.8%)	8 (44.4%)	96 (49.0%)	
> Male	78 (75.0%)	12 (16.2%)	10 (55.6%)	100 (51.0%)	
Smoking status, n (%)					0.001
> Current	16 (15.4%)	6 (8.1%)	8 (44.4%)	30 (15.3%)	
> Former or never	88 (84.6%)	68 (91.9%)	10 (55.6%)	166 (84.7%)	
Exacerbations in the past year*, n (%)					0.789
> Yes	36 (34.6%)	22 (29.7%)	6 (33.3%)	64 (32.7%)	
> No	68 (65.4%)	52 (70.3%)	12 (66.7%)	132 (67.3%)	
Hospitalization in the past year**, n (%)					0.100
> Yes	38 (36.5%)	16 (21.6%)	6 (33.3%)	60 (30.6%)	
> No	66 (63.5%)	58 (78.4%)	12 (66.7%)	136 (69.4%)	
Number of inhalers, n (%)					0.555
> 1	68 (65.4%)	54 (73.0%)	12 (66.7%)	134 (68.4%)	
> 2	36 (34.6%)	20 (27.0%)	6 (33.3%)	62 (31.6%)	
Inhaler type***, n (%)					
> DPI	65 (62.5%)	55 (74.3%)	17 (94.4%)	137 (69.9%)	0.039
> pMDI	9 (8.7%)	12 (16.2%)	5 (27.8%)	26 (13.3%)	0.119
> pMDI with VHC	21 (20.2%)	9 (12.2%)	0 (0.0%)	30 (15.3%)	0.076
> SMI	41 (39.4%)	21 (28.4%)	3 (16.7%)	65 (33.2%)	0.039
TAI (score)	48.9 ± 2.6	48.1 ± 4.2	47.2 ± 2.7	48.4 ± 3.3	0.089
Adherence by TAI, n (%)					0.069
> Good	70 (67.3%)	42 (56.8%)	6 (33.3%)	118 (60.2%)	
> Intermediate	26 (25.0%)	22 (29.7%)	8 (44.5%)	56 (28.6%)	
> Poor	8 (7.7%)	10 (13.5%)	4 (22.2%)	22 (11.2%)	
PDC (score)	82.2 ± 21.5	70.3 ± 25.3	67.6 ± 20.8	76.4 ± 23.7	0.001
Adherence by PDC, n (%)					0.001
> Good	70 (67.3%)	32 (43.2%)	6 (33.3%)	108 (55.1%)	
> Poor	34 (32.7%)	42 (56.8%)	12 (66.7%)	88 (44.9%)	
Inhaler visual identification, n (%)					0.073
> Correct	90 (86.5%)	70 (94.6%)	14 (77.8%)	174 (88.8%)	
> Incorrect	14 (13.5%)	4 (5.4%)	4 (22.2%)	22 (11.2%)	
Inhaler naming, n (%)					0.002
> Correct	32 (30.8%)	42 (56.8%)	8 (44.4%)	82 (41.8%)	
> Incorrect	72 (69.2%)	32 (43.2%)	10 (55.6%)	114 (58.2%)	

COPD, chronic obstructive pulmonary disease; DPI, dry powder inhaler; n, number; PDC, proportion of days covered; pMDI, pressurized metered-dose inhaler; SMI, soft mist inhaler; TAI, test of adherence to inhalers; VHC, valved holding chamber. *Due to their respiratory condition, without the need for hospitalization. **Due to their respiratory condition. ***Note that 62 (31.6%) participants had 2 inhalers.

Regarding the TAI score, a mean difference of 1.62 ± 0.67 was observed between patients with COPD and those with asthma+COPD (p=0.017). As for the PDC, the mean difference between patients with COPD and those with asthma was 11.95 ± 3.62 (p=0.001), and 14.62 ± 5.46 between patients with COPD and those with asthma+COPD (p=0.008).

A mild, positive linear correlation was found between TAI scores and PDC (r=0.37; p=0.000). 

The overall concordance between adherence categorization by the TAI score and PDC was 64.3%. Specifically, for COPD, asthma, and asthma+COPD, the concordance rates were 75.0%, 51.3%, and 55.5%, respectively.

Adherence to chronic inhalation therapy based on inhaler visual identification and naming is shown in Table 2.

**Table 2:** Characterization of adherence to chronic inhalation therapy according to inhaler visual identification and inhaler naming.

Adherence methods	Inhaler Visual Identification		p-value	Inhaler Naming		p-value
	Correct (n = 174)	Incorrect (n = 22)		Correct (n = 82)	Incorrect (n = 114)	
TAI (score)	48.6 ± 3.3	46.8 ± 3.4	0.017	48.2 ± 4.4	48.6 ± 2.2	0.398
Adherence by TAI, n (%)			0.000			0.009
> Good	110 (63.2%)	8 (36.4%)		56 (68.3%)	62 (54.4%)	
> Intermediate	50 (28.7%)	6 (27.2%)		14 (17.1%)	42 (36.8%)	
> Poor	14 (8.1%)	8 (36.4%)		12 (14.6%)	10 (8.8%)	
PDC (score)	78.1 ± 22.6	62.9 ± 28.0	0.004	78.5 ± 23.9	74.9 ± 23.6	0.293
Adherence by PDC, n (%)			0.061			0.161
> Good	100 (57.5%)	8 (36.4%)		50 (61.0%)	58 (50.9%)	
> Poor	74 (42.5%)	14 (63.6%)		32 (39.0%)	56 (49.1%)	

n: number; PDC: proportion of days covered; TAI: test of adherence to inhalers.

Tables 3 and 4 present the fractional logit model coefficients of adherence to chronic inhalation therapy, as measured by the PDC and TAI, respectively.

**Table 3:** Predictors of adherence to chronic inhalation therapy when measured based on PDC according to the results of the fractional logit model.

Determinant	Forest Plot 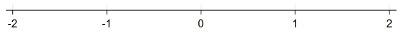	Coefficient (Std. Error)	z-value	p-value
Older age (≥ 65)	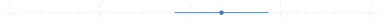	.3482674 (.2645803)	1.32	0.188
Sex (male)	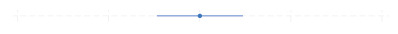	.0021043 (.2409677)	0.01	0.993
Smoking	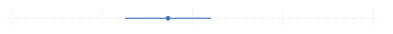	-.2712544 .2421464	-1.12	0.263
Pathology (Reference: Asthma)				
> COPD	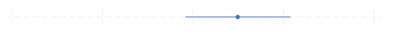	.4942129 (.2966214)	1.67	0.096
> Asthma+COPD	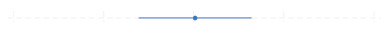	.0160121 (.3202303)	0.05	0.960
Inhaler visual identification	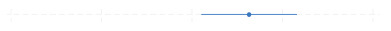	.6300489 (.2703577)	2.33	0.020
Inhaler naming	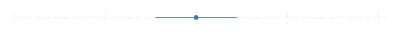	-.0040883 (.2277411)	-0.02	0.986
Multiple inhalers	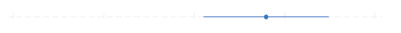	.7967108 (.3536215)	2.25	0.024
Exacerbations*	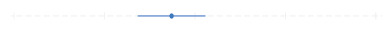	-.2570593 (.1916981)	-1.34	0.180
Hospitalizations^**^	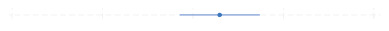	.2965935 (.2265943)	1.31	0.191
Inhaler type:				
> pMDI	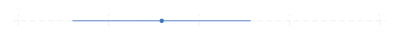	-.4136654 (.5035871)	-0.82	0.411
> pMDI with VHC	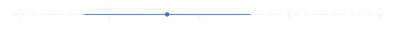	-.3607462 (.4723194)	-0.76	0.445
> DPI	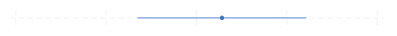	.2827291 (.476611)	0.59	0.553
> SMI	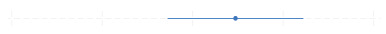	.4727223 (.3822675)	1.24	0.216
Constant	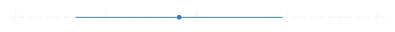	-.193884 (.5833687)	-0.33	0.740

COPD, chronic obstructive pulmonary disease; DPI, dry powder inhaler; PDC, proportion of days covered; pMDI, pressurized metered-dose inhaler; SMI, soft mist inhaler; Std, Standard; VHC, valved holding chamber. * Without the need for hospitalization. ^**^Due to their respiratory condition.

**Table 4:** Predictors of adherence to chronic inhalation therapy when measured based on TAI according to the results of the fractional logit model.

Determinant	Forest Plot 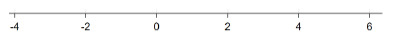	Coefficient (Std. Error)	z-value	p-value
Older age (≥ 65)	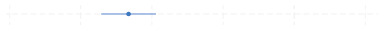	-.6564047 (.3945885)	-1.66	0.096
Sex (male)	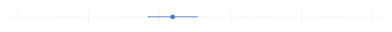	.3700067 (.3611608)	1.02	0.306
Smoking	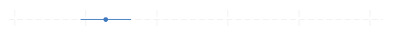	-1.436869 (.3632019)	-3.96	0.000
Pathology (Reference: Asthma)				
> COPD	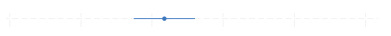	.3490573 (.4392229)	0.79	0.427
> Asthma+COPD	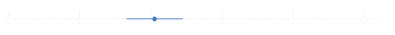	.1058628 (.4036983)	0.26	0.793
Inhaler visual identification	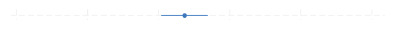	.7310443 (.3349092)	2.18	0.029
Inhaler naming	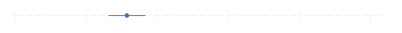	-.8541548 (.2669214)	-3.20	0.001
Multiple inhalers	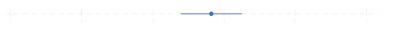	1.639927 (.4396881)	3.73	0.000
Exacerbations*	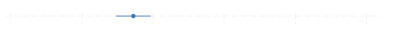	-.5519098 (.2504454)	-2.20	0.028
Hospitalizations**	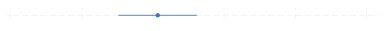	.1299456 (.5632743)	0.23	0.818
Inhaler type:				
> pMDI	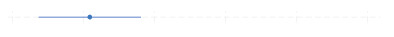	-1.81924 (.73418)	-2.48	0.013
> pMDI with VHC	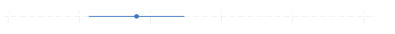	-.3884083 (.6841373)	-0.57	0.570
> DPI	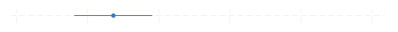	-1.279668 (.5610579)	-2.28	0.023
> SMI	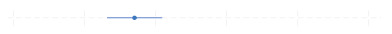	-.5880969 (.3978188)	-1.48	0.139
Constant	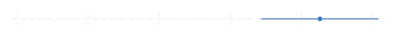	4.524437 (.8408395)	5.38	0.000

COPD, chronic obstructive pulmonary disease; DPI, dry powder inhaler; pMDI, pressurized metered-dose inhaler; SMI, soft mist inhaler; TAI, test of adherence to inhalers; VHC, valved holding chamber. * Without the need for hospitalization. ** Due to their respiratory condition.

The sensitivity, specificity, and area under the receiver operating characteristic (AUROC) curve for incorrect inhaler visual identification in detecting poorly adherent patients were calculated based on adherence classifications from the PDC and TAI scores. The results are shown in Table 5.

**Table 5:** Sensitivity, specificity, and AUROC of incorrect inhaler visual identification for detecting poorly adherent patients.

Adherence Assessment Method	Sensitivity	Specificity	AUROC
Proportion of Days Covered (PDC)	15.9%	92.6%	0.54
Test of Adherence to Inhalers (TAI) Score	17.9%	93.2%	0.56

AUROC, area under the receiver operating characteristic; PDC, proportion of days covered; TAI, test of adherence to inhalers.

## DISCUSSION

According to our findings, patients with COPD demonstrated significantly better adherence to inhaled therapy compared to those with asthma or asthma+COPD. Moreover, visual identification of inhalers was more strongly associated with adherence than correct naming and may serve as a useful tool for identifying patients with poor adherence.

The higher adherence rate among COPD patients compared to those with asthma is consistent with findings reported in previous studies.^3^ ^,^ ^20^ ^,^ ^21^ This may be partly age-related, as COPD patients in our study were significantly older than asthma patients (mean age: 72.1 vs 58.5 years). Older individuals may adhere more consistently to medication use due to increased health awareness, more frequent contact with healthcare providers, or fear of disease progression. Although age alone was not statistically significant in predicting adherence in the PDC model (p=0.188), it approached significance in the TAI model (p=0.096). When the individual variables age and pathology were replaced with an interaction term, the analysis revealed that older patients with COPD tend to exhibit better adherence than their younger counterparts-a relationship that warrants further investigation. While disease severity likely influences adherence, our study did not address this factor, highlighting yet another area for future research. The significant differences observed in adherence across the diagnostic groups underscore the need for tailored interventions to improve inhaler use and therapeutic compliance.

The concordance between adherence categorization by the TAI score and PDC was significantly higher in COPD patients (75.0%) than in those with asthma (51.3%) or asthma+COPD (55.5%). Additionally, poor adherence was more frequently identified using the PDC than the TAI score across all groups. These results suggest that asthma patients may overestimate their adherence to inhaled therapy to a greater extent than COPD patients when completing self-report questionnaires. The authors propose that at least two factors may help explain this discrepancy: 1) asthma patients tend to have lower adherence rates overall, which could lead to a wider gap between self-reported and objectively measured adherence; and 2) asthma is inherently a variable disease, with fluctuations in both severity and symptomatology that may lead to irregular inhaler use and influence patients’ perception of their adherence based on current symptom control. As a result, self-report questionnaires like TAI may yield more reliable adherence estimates in COPD patients than in those with asthma.

This study examined the relationship between patients’ ability to visually identify their inhaler(s) and adherence outcomes. Our findings indicate that correct visual identification is associated with better adherence, as measured by both the PDC and the TAI questionnaire. These results underscore the importance of patient education in adherence management. Ensuring that patients can visually recognize and correctly use their inhaler(s) should be a key component of strategies aimed at improving adherence.

Conversely, the ability to recall the commercial name or pharmaceutical components of an inhaler was not associated with better adherence when measured by the PDC and was even linked to poorer adherence when assessed using the TAI score. This may be partially explained by the picture superiority effect (PSE)-a cognitive phenomenon in which images are more easily recognized and remembered than names or labels.^22^ ^-^ ^24^ This finding may be particularly relevant for patients managing multiple medications, as visual cues can help reduce confusion and promote correct inhaler use. Therefore, recalling an inhaler’s name does not appear to be a reliable indicator of adherence and may not be a useful focus in adherence-improvement strategies.

The use of two inhalers, compared to a single inhaler, was associated with better adherence, regardless of whether it was measured using the TAI questionnaire or the PDC-an unexpected finding that contrasts with previous research.^25^ ^-^ ^29^ However, this result should be interpreted with caution considering the study’s design and population characteristics. Evaluating the association between the number of inhalers and adherence was not a predefined objective. Additionally, previous studies focus primarily on patients with COPD, who tend to be more adherent, whereas our sample included a mixed population of patients with asthma, COPD, and asthma+COPD. Prior research also frequently compared single-inhaler triple therapy with multiple-inhaler triple therapy, while in our study, single-inhaler therapies could contain one, two, or three components. Furthermore, most asthma patients-who typically exhibit lower adherence-used a single inhaler (73.0%). A more detailed analysis showed that 66.7% of asthma patients using a single inhaler had poor adherence based on the PDC, compared to only 30.0% of those using two inhalers.

Although the use of multiple inhalers appeared to be associated with better adherence in our study, another crucial factor to consider is inhaler technique. Previous studies have shown that using multiple inhalers is strongly associated with a higher risk of critical errors in inhaler technique, which can compromise clinical outcomes by reducing therapeutic efficacy.^30^ ^,^ ^31^ Therefore, we advocate for therapy simplification and recommend avoiding the use of multiple devices whenever possible, in accordance with current GOLD and GINA guidelines.^1^ ^,^ ^2^

Four additional determinants were found to be associated with adherence; however, they were only relevant when adherence was measured using the TAI, not the PDC. As such, the following findings should be interpreted with caution. The occurrence of exacerbations in the previous year (not requiring hospitalization) was associated with poorer adherence, consistent with findings from other studies.^25^ ^,^ ^32^ Smoking was also linked to lower adherence, although the literature on this association is mixed-some studies support our findings,^30^ ^,^ ^33^ while others have reported a positive association between smoking and adherence.^15^ In addition, the use of a dry powder inhaler (DPI) or a pressurized metered-dose inhaler (pMDI) without a valved holding chamber (VHC) was associated with poorer adherence. This may be partially explained by challenges patients face in coordinating actuation with inhalation (for pMDIs) or generating sufficient inspiratory flow (for DPIs), which can lead to frustration or a perceived lack of efficacy. 

According to the analyzed data, correct visual identification of the inhaler does not definitively classify a patient as adherent or non-adherent; however, it is associated with a greater likelihood of adherence. In such cases, clinicians should use additional methods to further assess adherence.

Using incorrect visual identification of inhalers to detect poorly adherent patients demonstrated low sensitivity but high specificity (93.2% with the TAI and 92.6% with the PDC). These results suggest that, overall, this method is not effective as a general screening tool. However, when a patient incorrectly identifies their inhaler(s), it serves as a reliable indicator of poor adherence.

Therefore, given its rapid and practical application, the authors believe that the image set proposed in this study**-which** encompasses all inhalation devices available on the national market**-**could serve as a valuable tool in clinical practice, particularly for identifying patients with poor adherence. It should also be considered as part of a broader adherence assessment strategy.

The main limitations of this study include its single-center design, the cross-sectional nature of some data, and the partial reliance on self-reported information. Furthermore, the image set used reflects inhalation devices available in our national market, which may not be representative of those found in other countries. Key strengths of the study include its prospective design, the incorporation of a visual identification component**-an aspect not** previously explored in other studies**-**and the use of multiple adherence assessment methods, providing a comprehensive evaluation from both subjective and objective perspectives.

In conclusion, asthma patients appear to overestimate their adherence more than COPD patients when completing self-report questionnaires. The ability to visually identify inhalers was associated with better adherence, whereas the ability to recall the commercial name or pharmaceutical components was not. Although incorrect visual identification has limited value as a general screening tool, it can still reliably identify poorly adherent patients, providing a quick and practical strategy for use in clinical practice.

Future studies should explore broader assessment methods and aim to develop a tool that combines visual identification with other adherence metrics. Such a tool should be designed for rapid use during medical consultations to facilitate the early detection of poor adherence.
